# Incorporating a quiz into informed consent processes: Qualitative study of participants' reactions

**DOI:** 10.1186/1475-2875-6-145

**Published:** 2007-11-10

**Authors:** Sassy Molyneux, Caroline Gikonyo, Vicki Marsh, Philip Bejon

**Affiliations:** 1 Senior Research Fellow, Kenya Medical Research Institute Centre for Geographic Medicine Research Coast (KEMRI CGMRC) Wellcome Trust Research Programme, Kilifi, Kenya, P.O. Box 230; 2 Research Assistant, Kenya Medical Research Institute Centre for Geographic Medicine Research Coast (KEMRI CGMRC) Wellcome Trust Research Programme, Kilifi, Kenya, P.O. Box 230; 3 Senior Scientist, Kenya Medical Research Institute Centre for Geographic Medicine Research Coast (KEMRI CGMRC) Wellcome Trust Research Programme, Kilifi, Kenya, P.O. Box 230; 4 Clinician and Senior Research Fellow, Centre for Clinical Vaccinology and Tropical Medicine, University of Oxford, Churchill Hospital, Oxford, UK

## Abstract

**Background:**

Formal checks of participant understanding are now widely recommended to improve informed consent processes. However, the views of the participants these assessments are designed to protect are rarely considered. In this paper the findings of a qualitative study aimed at documenting community reactions to a semi-structured questionnaire ('quiz') are reported. The quiz was administered to 189 mothers after consenting for their children to participate in a malaria vaccine trial on the Kenyan Coast.

**Methods:**

Once the malaria vaccine trial was underway, focus group discussions were held with some of these mothers (nine groups; 103 mothers), and with community-based field staff attached to the malaria vaccine trial (two groups of five workers). Individual interviews with other trial staff were also held.

**Results:**

The quiz prompted community members to voice concerns about blood sampling and vaccine side-effects, thereby encouraging additional discussions and interactions between the research team and potential study participants. However, it also caused significant upset and concern. Some of the quiz questions, or the way in which they were asked, appeared to fuel misconceptions and fears, with potentially negative consequences for both the study and community members.

**Conclusion:**

Formal approaches to checking study understanding should be employed with sensitivity and caution. They are influenced by and impact upon complex social relationships between and among researchers and community members. Adequate consideration of these contexts in assessments of understanding, and in responding to the issues raised, requires strong social science capacity.

## Background

Individual informed consent is a key ethical obligation for clinical studies. However, empirical studies show that the key requirements are often not met [1-5]. Suggestions for improving informed consent abound [2-12], but few have been evaluated in the field.

There is some evidence that a formal assessment, administered during the informed consent process, improves understanding[4]. Checks of study comprehension, often in the form of a simple quiz with a check-list of facts, are, therefore, increasingly recommended for clinical studies[10,13,14]. In a recently published Nuffield discussion document, the monitoring of participants' understanding by a separate team was described as a '*valuable addition to many trials conducted in developed countries, where participants may have an incomplete understanding of the implications of their participation' *[14]. The need to assess participants' general understanding of the implications of the trial, rather than to simply test their retention of information with a check-list, was emphasized.

**Figure 1:**
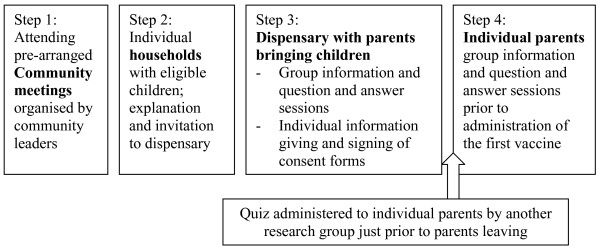
Formal information giving processes organised by vaccine trial team.

In this paper, community reactions to a semi-structured questionnaire aimed at exploring participant perceptions and understanding of a malaria vaccine trial are described. The trial was conducted at the KEMRI-Wellcome Trust Collaborative Research Programme on the Kenyan Coast. The trial, information giving processes, and community perceptions and understanding of the trial itself are described in detail elsewhere[15,16]. Briefly, information-giving included: creating awareness of the study among community representatives and the general community at public meetings, and then with parents in their own homes; holding detailed group and individual discussions with parents who volunteered to attend the local dispensary to enrol their children in the trial; and a further explanation of the study at least a week after signing consent, just prior to administration of the vaccine (Figure 1). The findings of the questionnaire (or quiz) are briefly reported here. The unexpected reactions to the quiz are then described in more detail, in order to inform appropriate consenting processes in similar communities. This research was part of a protocol reviewed and approved by the Kenyan National Ethical Review Committee (No. 925).

## Methods

The Social and Behavioural Research (SBR) group coordinate community liaison activities at the KEMRI-Wellcome Trust Research Programme. SBR members (SM, CG and VM) adapted a quiz passed by the local and national ethics review committees into a semi-structured questionnaire (hereafter 'quiz') administered to 189 caretakers, primarily mothers (see Additional file 1). The quiz was administered by four experienced SBR fieldworkers, all of whom were from the same locality as the participants and had been carefully trained in communication skills. To avoid disrupting consenting and clinical screening processes, caretakers were interviewed just prior to their departure from the dispensary on the screening day (Step 3; Figure 1). Community reactions to the quiz itself were initially raised by the community to the trial field staff and PI (PB) and were then explored by CG through focus group discussions with community members (n = 9) and fieldworkers (n = 2) once the trial was underway. Community members were mothers of children in the trial, selected on the basis of village of residence. Focus Group Discussions (FGDs) continued to be held until a point of redundancy was reached (no new information being presented in the groups).

## Results and Discussion

The proportion of correct responses regarding different key study details ranged from 29% to 85% (Table 1), and the proportion responding 'don't know' from 14% to 45%. 35% of participants responded to the question 'what is this work for' with a correct response. Most participants providing an 'incorrect' response described vaccine or malaria prevention work in general terms, without specifying testing for safety or potential efficacy of a malaria vaccine. In subsequent FGDs, and in responses to other questions, it appeared that many more parents did in fact understand that the study was a trial, and that the problem was primarily with the question and it's administration rather than with participant knowledge. For the remaining questions, 'incorrect' responses included 6% (n = 11) reporting that all children would be given a malaria vaccine, 18% (n = 35) that their child would receive one or two rather than three injections, and 20% (n = 39) that children with the vaccine would have assured protection against malaria infection.

**Table 1:** Correct and don't know answers on key vaccine trial study information

**Question**	Correct response %	Stating DK %
What is this research for?	35	26
Why vaccine can't be given to all Kenyan children at the moment?	48	45
If you're child is healthy, and take part, how many injections will they receive?	49	31
Will all children be given a malaria vaccine?	85	16
Why will you have to stay at the clinic for one hour after the injection	80	14
Will children who have received a vaccine be able to get malaria?	29	44

No concerns were openly vocalized about the quiz itself while it was being administered. However, over the course of quiz one mother became increasingly agitated and finally withdrew from the study, demanding that her child's blood sample be returned and destroyed to prevent 'devil-worshipping'. Many other potential participants observed this dramatic and public event. Discussions around it revealed that rumours were widespread in the community: rumours that the information given by the research team is a lie; that unnecessarily large samples of blood are taken to be sold or given to devils (often associated with 'wazungus', or foreigners); that children will fit and die immediately after the vaccine is administered; that children will slowly die off after the end of the vaccine trial; and that biscuits and juice are given to children to increase their blood for further blood taking for devils.

Recognition of the huge sensitivity around blood sampling led to public meetings at which the PI showed and answered questions about how blood is processed and used in the laboratory; including demonstrations of microscope slides and assay wells. These meetings were an opportunity to focus on an area that had not been covered in depth in previous information giving sessions. More importantly, they were an additional opportunity for community members to test the research teams' openness and consistency in messages. These are behaviours supportive of trust-building, and appeared essential to the successful conduct of the trial (see also Gikonyo *et al *[16]).

**Figure 2:**
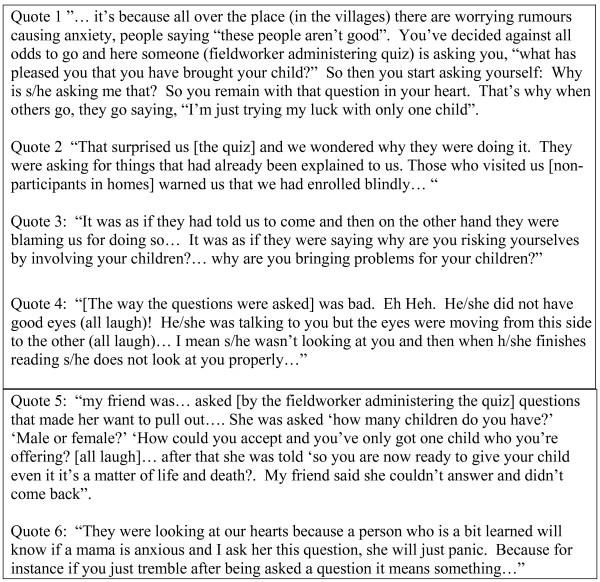
Examples participants' concerns about the quiz.

Over the course of public meetings and in other informal interactions, concerns about the quiz itself began to emerge. Questions about the quiz were, therefore, included in FGDs with fieldworkers and community members. In almost all FGDs, participants mentioned at least one disadvantage of the quiz. An important view expressed in community groups was that the questions asked, and the way they were asked, heightened concerns about rumours circulating in the community; and that raising concerns in this way was unnecessary (see above event, and Quotes 1–3; Figure 2). Further concerns included: people were not given a specific explanation of the quiz in previous information giving steps and so were not prepared for it; a person was being taken 'very far and interviewed alone'; and possible poor verbal and non-verbal communication by interviewers (Figure 2, quote 4). Reports of questions asked suggest that either the questions were being confused with a larger census that is on-going in the area, or that some of the interviewers embellished the questions and contributed to the concerns raised (Figure 2; quote 5). Participants' concerns about the questions may have led to a relatively high level of 'don't know' responses to the quiz, either in protest at the questions, or in an attempt to gather additional information from SBR fieldworkers to cross-check vaccine trial team information.

**Figure 3:**
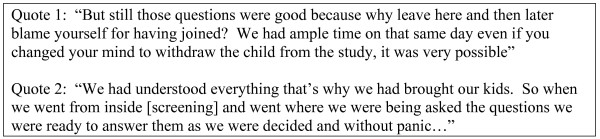
Examples of the participants' positive comments about the quiz.

In six FGDs participants mentioned at least one positive aspect of the quiz, including that it helped people understand, gave them a chance to seek clarification on specific issues (from the study team or friends and relatives), and assisted in decision-making about study participation (Figure 3; Quotes 1 and 2). Those who reacted badly were sometimes reported not to have understood, and any concerns raised were reportedly resolved once the trial began. 

Nevertheless, the quiz was clearly an unnerving experience for some participants (Figure 2; Quote 6), particularly given the relatively low education level of many mothers compared with the field assistants interviewing them. One person wondered what good the quiz did for those who decided not to join, because many of the rumours circulating in the community were proven untrue (for example children did not die immediately after receiving the first vaccine), and because those not in the trial did not benefit from careful monitoring and free treatment: P: (Interrupted a discussion on the positive aspects of the quiz)... *it helped some; those who decided to continue coming. But then those that misunderstood, it didn't help them because they ran away! So, did they finish the journey?*

## Conclusion

Formal approaches to checking study understanding are increasingly popular for clinical research. The findings presented in this paper suggest that any such checks should be employed with sensitivity and caution. In the absence of other studies that have formally explored this issue, the experiences in Kenya suggest the following key issues influencing community reactions and the validity of the quiz itself:

The interviewer. The value of quizzes is dependent on the interpersonal skills of the people administering it, and their ability to ask questions in an open and non-threatening way. Difficulties were faced in Kenya even with fieldworkers fluent in the local language who were carefully trained in qualitative methodologies and in communication skills. Interviewers from within the team may have been more appropriate in this case.

Qualitative versus quantitative data. The findings of quantitative data acquired can be misleading without complementary qualitative data. Using quiz data alone to make definitive comments on individual levels of understanding or eligibility to access a trial is not therefore recommended. Imbalances in resources and research knowledge between researchers and community members can also make individual interviews intimidating for participants. In Kenya, a qualitative, group based, or less formal quiz may well have achieved the same positive impacts for all with fewer concerns raised for community members.

The follow up provided after a quiz. A formal check of understanding can lead to difficulties for individuals, for the community and for researchers in the short term. Nevertheless, this is an opportunity for the issues raised to be dealt with, leading to better understanding on both sides, and a stronger relationship between participants and researchers. This is particularly the case if the assessment is incorporated into and informs a broader strategy for involving communities in aspects of the research.

Expertise is required. Developing, administering and responding to assessments of understanding for clinical trials requires significant social science capacity. Strengthening the role of good quality social science in clinical trials is an important strategy for improving ethical practice.

The findings may be specific to the study population and to the socio-cultural and institutional context of the study. Use of a similar quiz in phase 1 trials, on a much smaller sample size of well-educated parents, provoked less controversy. However this was not formally explored. Further studies on quizzes would indicate the generalizability of the findings and identify important contextual influences. A greater understanding of community perspectives on quizzes, and of other ethical requirements, will help give research participants and communities a voice in national and international debates on how to strengthen ethical practice.

## Authors' contributions

All authors were involved in the design and conduct of the study, data analysis, and the preparation and review of the manuscript. Caroline Gikonyo was responsible for data collection. Sassy Molyneux had full access to all of the data in the study and takes responsibility for the integrity of the data and the accuracy of the data analysis.

## Supplementary Material

Additional file 1 Appendix 1. The introduction to and questions covered in the quiz Click here for file

## References

[B1] Silverman AW (1989). The myth of informed consent: in daily practice and in clinical trials. Journal of Medical Ethics.

[B2] Bosk CL (2002). Obtaining voluntary consent for research in desperately ill patients. Med Care.

[B3] Lidz CW, Appelbaum PS (2002). The therapeutic misconception: problems and solutions. Med Care.

[B4] Fitzgerald DW, Marotte C, Verdier RI, Johnson WD, Pape JW (2002). Comprehension during informed consent in a less-developed country. Lancet.

[B5] Molyneux CS, Peshu N, Marsh K (2004). Understanding of informed consent in a low-income setting: three case studies from the Kenyan Coast. Soc Sci Med.

[B6] Agre P, Campbell FA, Goldman BD, Boccia ML, Kass N, McCullough LB, Merz JF, Miller SM, Mintz J, Rapkin B, Sugarman J, Sorenson J, Wirshing D (2003). Improving informed consent: the medium is not the message. Irb.

[B7] Agre P, Rapkin B (2003). Improving informed consent: a comparison of four consent tools. Irb.

[B8] Allmark P, Mason S, Gill AB, Megone C (2003). Obtaining consent for neonatal research. Arch Dis Child Fetal Neonatal Ed.

[B9] Edwards SJ, Lilford RJ, Thornton J, Hewison J (1998). Informed consent for clinical trials: in search of the "best" method. Soc Sci Med.

[B10] Kass NE, Maman S, Atkinson J (2005). Motivations, understanding, and voluntariness in international randomized trials. Irb.

[B11] Molyneux CS, Peshu N, Marsh K (2005). Trust and informed consent: insights from community members on the Kenyan coast. Soc Sci Med.

[B12] Smyth RL, Weindling AM (1999). Research in children: ethical and scientific aspects. Lancet.

[B13] Hyder AA, Wali SA (2006). Informed consent and collaborative research: perspectives from the developing world. Developing World Bioeth.

[B14] NCOB (2005). The ethics of research related to health care in developing countries:  a follow-up discussion paper..

[B15] Bejon P, Mwacharo J, Kai O, Mwangi T, Milligan P, Todryk S, Keating S, Lang T, Lowe B, Gikonyo C, Molyneux C, Fegan G, Gilbert SC, Peshu N, Marsh K, Hill AV (2006). A Phase 2b Randomised Trial of the Candidate Malaria Vaccines FP9 ME-TRAP and MVA ME-TRAP among Children in Kenya. PLoS Clin Trials.

[B16] Gikonyo C, Bejon P, Marsh V, Molyneux CS (2006). Taking social relationships seriously:  lessons learned from the informed consent practices of a vaccine trial on the Kenyan Coast. Social Science and Medicine.

