# Supplementary material for: Incorporating a quiz into informed consent processes: Qualitative study of participants' reactions
Source: Malar J. 2007 Nov 10;6:145. doi: 10.1186/1475-2875-6-145 (PMC2204020; doi:10.1186/1475-2875-6-145)
Supplement: Additional file 1 — Appendix 1. The introduction to and questions covered in the quiz [file 1475-2875-6-145-S1.doc]

## Appendix 1:

## Introduction:

## I work for another section of KEMRI where we are trying to learn how we can improve our explanations about our work. To help us do this I would like to ask you some questions about what you have learned about the work discussed today, where you learned it, and if there was anything you found worrying/confusing.

- Where have you learned about this work of KEMRI’s?
- Where have you learned most? What makes you say that?
- Overall what do you like about this work of KEMRI’s, and why?
- Any recommendations/questions?
- Why have you decided that your child should join/ should not join this study?

Can I find out in more detail what you have learned about this work?

- What is this research for?
- Why can the vaccine not be given to all Kenyan children at the moment?
- If you’re child is healthy, and take part, how many injections will they receive?
- Will all children be given a malaria vaccine? Y/N (If not why not)
- Why will you have to stay at the clinic for one hour after the injection
- Will children who have received a vaccine be able to get malaria? Y/N (explain)

Any other details you remember?
